# A New Perspective on Maternal Ill-health and Its Consequences

**DOI:** 10.3329/jhpn.v30i2.11293

**Published:** 2012-06

**Authors:** Mary Ellen Stanton, Neal Brandes

**Affiliations:** United States Agency for International Development, Washington, DC, USA

The purpose of the set of studies published in this issue of the *Journal of Health, Population and Nutrition* is to examine long- and short-term consequences of maternal complications for mothers and newborns and to document the physical, social, psychological and economic impacts of maternal ill-health and maternal and perinatal death on women and others living in the family unit. This work reflects the spirit of President Obama's Global Health Initiative that draws special attention to women- and girl-centred approaches as central to the advancement of health and development.

In the past, the documentation of the global estimates of reductions in maternal mortality has not been matched by more in-depth efforts to characterize and understand the continuing burden of maternal complications, morbidity, and disability suffered by childbearing women in developing countries. With the exception of recent more in-depth studies in India, Burkina Faso, and Benin, the global understanding of reduction in maternal mortality and morbidity is based upon the estimates of the number and proportion of childbearing women who die (1). By and large, the documentation of morbidities during or after the intrapartum period has lacked specificity and precision to inform the country and the programme managers on the incidence of immediate disabilities and of longer-term consequences for women, their families, and their communities. The grand syntheses are limited by available evidence—meagre compared to the magnitude and wide array of potential consequences of maternal ill-health on individuals and society (2,3).

This series of papers presents, for the first time in two geographic areas, a comprehensive snapshot of the short- and long-term consequences of acute maternal morbidity. The icddr,b surveillance site in Matlab, Bangladesh, has a unique set of records of the reproductive health of individual women that provide data accumulated for decades. This was selected as an ideal site to draw upon the database to examine retrospectively long-term and prospectively selected short-term consequences of maternal ill-health. This is the first attempt to obtain greater precision on the consequences of maternal ill-health, using a robust methodology and an extensive dataset, with added qualitative studies and postpartum physical examinations of women following childbirth. In addition, we have included a study that provides contrasting and additional information from Action Research and Training for Health in rural Rajasthan, India.

The Bangladesh study found that for every maternal death, there are about 40 severe/less-severe complications and over 160 postpartum morbidities/disabilities. These numbers are far higher than previous estimates of 20 women with complications and 40 with postpartum morbidities/disabilities for every maternal death (4).

In undertaking this work, it became apparent that even with the provision of free services and the capability to assess and manage the physical conditions of postpartum women, it is extremely hard to ensure that all who need services obtain them. As a result, it appears from the data that women from higher-income families have a higher burden of morbidity than poorer women. In reality, the poorer women remain outside the available services for various reasons, and their experiences are not reflected in the documentation.

## Immediate consequences of maternal complications

In Matlab, about 10% of women have a severe or less-severe maternal complication during the intrapartum period (Huda *et al.*). While severe dystocia is the most common complication, women are most likely to die of haemorrhage. Most women who died sought care from public or private facilities; about 25% died at home.

The Bangladesh study found that physical complications following delivery are common—over 40% of women, including those with acute maternal complications during the intrapartum period and those with normal vaginal births, suffered from some postpartum morbidity—but most are relatively mild, including first-degree uterine prolapse, haemorrhoids, and hypertension (Ferdous *et al.*).

## ‘Consequences of the consequences’

As part of this series of studies, a paper previously published (5) showed a substantial effect of the death of the mother on the survival of her children. “The cumulative probability of survival up to age 10 years was 24% in children whose mothers died before their tenth birthday compared to 89% in those whose mothers remained alive. The greatest effect was noted in children aged 2-5 months, whose mothers had died. The effect of the father's death on cumulative probability of survival of the child up to 10 years of age was negligible” (5).

An important finding is that infant mortality is approximately eight times higher for those infants whose mothers died than if the mother survived (5). This finding has enormous implications for our care for these infants who are now maternal orphans.

Beyond survival, the studies describe a vast array of sequelae following obstetric complications—some very serious and some less prevalent or more serious than we had anticipated. While there was a small effect of maternal anaemia on young children's language ability, there were often substantial consequences for women due to different morbidities (Hamadani *et al.*). Not surprisingly, there is a vast array of quality of life issues. For example, beyond the physical results of fistula, uterine prolapse, and incontinence, there is documentation of profound effects on women's daily activities. And, in the case of a perinatal death, women may be sequestered for years and be unable to even carry out religious rituals that they consider fundamental to their spiritual well-being (Khan *et al.*).  The consequences of a perinatal death on the mother include postpartum depression as well as emotional violence and controlling behaviour by the family and the community.

The study finds that there is a significant association between Bangladeshi women who report negative experiences with their childbirth and postpartum depression (Gausia *et al.*). Furthermore, women with conditions of chronic maternal morbidities, such as uterine prolapse, sometimes experience *khota* (insult) whereby they are ridiculed by neighbours and in-laws for jeopardizing the marriage through not meeting the sexual needs of the husband or not carrying out household responsibilities. Women described physical and sexual violence in response to not meeting husbands' demands. The subordinate role of women subjects them to an array of hardships and injustices that result from their chronic morbidities and disabilities (Khan *et al.*).

This research has elicited considerable detail about the economic consequences of maternal morbidity, which are the highest within the six weeks after birth and decline substantially by six months (Hoque *et al*.). Faced with maternal complications, families take loans and, to a lesser extent, sell assets to pay for healthcare. By interviewing cohorts of families, the study found that, even among the poorest households, there was unexpected resiliency to the economic shock of the cost of paying for obstetric emergencies. Families invest in their women in Bangladesh and will bypass lower-level facilities perceived to have lower quality to seek care for obstetric emergencies. These findings differ from findings in other country settings and point to the need for more robust methodologies and more comparable studies in other settings to increase the understanding of various coping strategies and both economic and non-economic consequences—beyond the actual financial debt.

## Evolving context in Bangladesh

We recognize that the environment in Bangladesh is highly dynamic. Maternal mortality declined by 40% to 194/100,000 livebirths between 2001 and 2010 (6). Death from maternal causes now follows cancers and circulatory diseases as the major causes of death of Bangladeshi women of reproductive age. This progress appears to result from improved awareness of the need for care during emergencies and overall increased care-seeking for delivery, higher levels of maternal education, better economic conditions, and reduction in fertility—the total fertility rate has fallen to 2.5 (6). As a result, we are not finding the extent of long-term injury apparently suffered by women in other settings, particularly in settings where maternal mortality is higher. With the lowering of fertility and maternal mortality and the increased use of services, there is the prospect that more complications leading to disabilities can be prevented.

## Comparing India and Bangladesh findings

The study in rural Rajasthan, India, published in this special issue, focused on the physical problems of postpartum women (Iyengar). As in Bangladesh (6), delivery-care in Rajasthan is rapidly moving to the health facility. Unlike in Bangladesh, moderate and severe anaemia is the most common maternal morbidity, followed by puerperal infections. Both the studies raise the concern that the postpartum period is one with high risk for women.

Given the variation in the pattern of postpartum maternal morbidities and disabilities in the Bangladesh and India studies, additional research in other country contexts is needed to quantify the changes in the burden of disease and to add to our knowledge concerning the many maternal morbidities and disabilities not now included in the calculation of disability-adjusted life-years.

## Recommendations

The results of this wide-ranging set of studies make the case for galvanizing attention in a Call to Action to highlight the importance of postpartum and postnatal care that goes beyond the standard 4-6-week period. This will involve communications to women and their families about the importance of postpartum care and assessing the risk and actual occurrence of complications and responding effectively to them by healthcare providers. It will also mean following up women who have had a normal vaginal delivery, especially those who remained at home. The children of mothers who died and the mothers of perinates who died require very special attention because of their increased vulnerability.

Screening is called for several times following the birth, whether inside or outside a healthcare facility since problems may emerge or be recognized at different times in the postpartum period. All women need to be screened for physical postpartum problems, including anaemia, infection, incontinence, uterine prolapse, and obstetric fistula, and be managed, along with the provision of family planning and counselling for taking care of themselves and their babies. Women also need to be screened for postpartum depression and experience of emotional, physical and sexual violence. There are obvious possibilities for making this happen—first, at the time of hospital discharge and at the ‘usual’ 4-6-week postpartum check-up, and perhaps at immunization points for the child. We need to assess and re-assess. The findings of this study in Matlab, Bangladesh, point to the need for follow-up, including outreach to the community, especially when the birth has occurred at home, and particularly to follow-up on the survivors of a maternal or perinatal death. These mothers and babies are highly vulnerable and require special attention.

Second, beyond screening, we need improvements in the healthcare system to build individual expertise and organizational capacity to respond to and effectively treat problems ranging from severe uterine prolapse and fistula, requiring specialized surgery, to depression and violence, requiring specialized counselling and intervention.

Third, social protection is vital for the most vulnerable. Although this study suggests innovative coping and care-seeking strategies by Bangladeshi families, an effective health system is needed to provide accessible preventive and lifesaving care, particularly for the most economically- and socially-vulnerable populations.

Finally, since societal customs and expectations set the stage for perpetrating and condoning the devastating emotional, physical and sexual abuse that women experience as a result of chronic morbidities and disabilities, these problems will not all be solved by healthcare providers or health services improvement alone. We must spotlight the fundamental issues of status and human rights of women for which the answers will be found in education, employment, and empowerment of women, in partnership with men, to improve the well-being of families.

## ACKNOWLEDGEMENTS

The opinions expressed in this paper are those of the authors and not necessarily of the United States Agency for International Development where they work.
